# Inhibition of MHC Class I Is a Virulence Factor in Herpes Simplex Virus Infection of Mice

**DOI:** 10.1371/journal.ppat.0010007

**Published:** 2005-09-30

**Authors:** Mark T Orr, Kurt H Edelmann, Jeffrey Vieira, Lawrence Corey, David H Raulet, Christopher B Wilson

**Affiliations:** 1 Department of Immunology, University of Washington, Seattle, Washington, United States of America; 2 Department of Laboratory Medicine, University of Washington, Seattle, Washington, United States of America; 3 Program in Infectious Diseases, Fred Hutchinson Cancer Research Center, Seattle, Washington, United States of America; 4 Department of Medicine, University of Washington, Seattle, Washington, United States of America; 5 Department of Molecular and Cell Biology and Cancer Research Laboratory, University of California, Berkeley, California, United States of America; 6 Department of Pediatrics, University of Washington, Seattle, Washington, United States of America; University of California at San Francisco, United States of America

## Abstract

Herpes simplex virus (HSV) has a number of genes devoted to immune evasion. One such gene, ICP47, binds to the transporter associated with antigen presentation (TAP) 1/2 thereby preventing transport of viral peptides into the endoplasmic reticulum, loading of peptides onto nascent major histocompatibility complex (MHC) class I molecules, and presentation of peptides to CD8 T cells. However, ICP47 binds poorly to murine TAP1/2 and so inhibits antigen presentation by MHC class I in mice much less efficiently than in humans, limiting the utility of murine models to address the importance of MHC class I inhibition in HSV immunopathogenesis. To address this limitation, we generated recombinant HSVs that efficiently inhibit antigen presentation by murine MHC class I. These recombinant viruses prevented cytotoxic T lymphocyte killing of infected cells in vitro, replicated to higher titers in the central nervous system, and induced paralysis more frequently than control HSV. This increase in virulence was due to inhibition of antigen presentation to CD8 T cells, since these differences were not evident in MHC class I-deficient mice or in mice in which CD8 T cells were depleted. Inhibition of MHC class I by the recombinant viruses did not impair the induction of the HSV-specific CD8 T-cell response, indicating that cross-presentation is the principal mechanism by which HSV-specific CD8 T cells are induced. This inhibition in turn facilitates greater viral entry, replication, and/or survival in the central nervous system, leading to an increased incidence of paralysis.

## Synopsis

While animal models are often instructive in understanding human diseases, many factors that influence disease differ between mouse and man. Although mice can be experimentally infected with HSV-1, this virus has evolved as a human pathogen. One facet of this evolution is HSV's mechanisms to evade the immune response, allowing the virus to persist for the lifetime of the human host. This evasion includes preventing CD8 T cells from recognizing and killing infected cells by inhibiting the expression of the molecule that presents viral peptides to CD8 T cells: major histocompatibility complex (MHC) class I. HSV is unable to inhibit mouse MHC class I, thus rendering this immune-evasion strategy inoperative in the mouse. To better understand the biology of HSV infection and the immune response to this virus in humans, the authors corrected this deficiency by inserting a gene which inhibits murine MHC class I. This recombinant virus demonstrates that MHC class I inhibition is an important determinant of disease progression. The authors found that the recombinant HSV still effectively elicits a CD8 T-cell response, but this response is ineffective in controlling the infection. This finding reveals the important distinction between the size of the immune response and the effectiveness of the response, which may be important to HSV vaccine studies.

## Introduction

Herpesviruses are distinguished by their ability to establish lifelong infection cycling between lytic and latent phases. One challenge to this lifestyle is that the immune system of the vertebrate hosts has the opportunity to be repeatedly primed, thereby increasing the potential for the host to eradicate the pathogen. To cope with this challenge, herpesviruses have evolved multiple mechanisms to evade immune detection or clearance. These mechanisms target all aspects of the immune response, including antibodies, chemokines, cytokines, natural killer (NK) cells, and CD4 and CD8 T cells [1,2].

Major histocompatibility complex (MHC) class I molecules are a particularly attractive target for immune evasion by viruses, because decreasing expression and/or antigen presentation by MHC class I can attenuate CD8 T-cell-mediated recognition of infected cells [3–5]. Inhibition of MHC class I antigen presentation is a hallmark of the herpesvirus family with all family members having at least one mechanism to achieve this. For example, the murine cytomegalovirus (MCMV) m152 gene product gp40 binds to the MHC class I/peptide complex in the ER/*cis*-Golgi compartment preventing export to the cell surface [6,7]. The human cytomegalovirus (HCMV) US11 gene product binds nascent MHC class I heavy chain in the endoplasmic reticulum and targets it to the cytosol for proteasomal degradation [8,9]. However, any strategy that lowers surface expression of MHC class I carries with it the undesirable (from the perspective of the virus) inverse effect of reducing the inhibitory signal that MHC class I exerts on NK cell activation. Some herpesviruses compensate for this decrease in MHC class I by expressing proteins that inhibit cell-surface expression of ligands that activate NK cells [10]. For example, MCMV m152 inhibits expression of ligands for the activating NK cell receptor NKG2D, while MCMV m157 binds to members of the Ly49 family of NK cell receptors, which include both inhibitory and activating receptors [11–13].

Herpes simplex virus (HSV) is an α-herpesvirus that establishes lifelong infection in neuronal cells from which it periodically reactivates [14]. Like the β-herpesviruses MCMV and HCMV, HSV inhibits antigen presentation on MHC class I to CD8 T cells, having evolved two distinct mechanisms by which to do so: the viral host shutoff protein (vhs) and the immediate early US12 gene product ICP47. Vhs targets host mRNA for destruction, thus nonspecifically shutting down antigen presentation at several steps [15,16]. ICP47 directly targets MHC class I antigen presentation by binding to the transporter associated with antigen presentation (TAP) 1/2 complex, preventing transport of peptides from the cytosol to the endoplasmic reticulum where peptides are loaded into the nascent MHC class I heavy-chain β_2_ microglobulin (β_2_m) complex [17–19]. However, genes that would compensate for the agonistic effect of reduced MHC class I expression on NK cell activation have not been identified in HSV.

Although HSV has no known murine homolog, HSV can infect mice in experimental models. In mice, as in humans, HSV spreads from peripheral tissues to the dorsal root ganglia in which it can establish latency or from which it can spread to the central nervous system (CNS) producing paralysis and death [20]. Consequently, murine models have been used extensively to study the pathogenesis and immunological control of HSV infection. One limitation of current murine models of HSV infection is that ICP47 poorly inhibits TAP in mouse cells [21]. This is due to ~100-fold decreased binding of ICP47 to murine TAP1/2 as compared to human TAP1/2 [18]. Consistent with this, ICP47 protects HSV-infected human fibroblasts from destruction by cytotoxic T lymphocytes (CTLs), while murine fibroblasts are not protected [22]. Despite the limited ability of ICP47 to inhibit murine TAP, a role for ICP47 in evasion of CD8 T-cell-mediated immunity in mice was suggested by studies in which CD8 T cells were able to protect mice from a mutant HSV lacking ICP47 but not from wild-type virus [23]. Given the difference in the capacity of ICP47 to inhibit peptide loading by murine versus human TAP, the contribution of CD8 CTL to HSV immunity in mice may overestimate their role in control of HSV in humans.

To develop an experimental system in which inhibition of MHC class I by HSV in mice would more closely parallel the situation in humans, we generated recombinant herpes simplex viruses (rHSVs) expressing MCMV m152 or HCMV US11. Both of these proteins inhibit antigen presentation by murine MHC class I [24,25]. We report here that these rHSVs prevented CTL recognition of infected cells in vitro, resulted in increased viral burden in the CNS, and increased the frequency of paralysis-induction compared to mice infected with control HSV. By contrast, these differences were not observed in MHC class I-deficient mice or in mice in which CD8 T cells were depleted. The generation of HSV-specific CD8 T cells was not affected, suggesting that the greater pathogenicity of these viruses resulted from evasion of CD8 T-cell recognition in the CNS, not impaired priming of the adaptive immune response.

## Results

### rHSVs Are Generated

**Figure 1:**
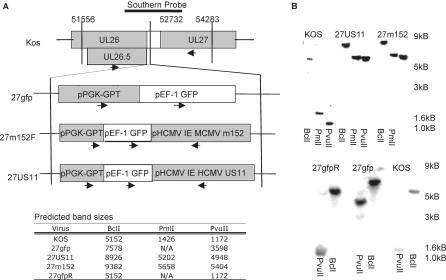
Generation of rHSVs (A) rHSVs expressing MCMV m152 (27m152), HCMV US11 (27US11), or only the selection cassette (27gfp) were generated via homologous recombination with KOS strain HSV-1. Contents and location of insertions are indicated. Arrows indicate direction of transcription. The probe used to isolate correctly recombined viruses is indicated. (B) Genomic DNA from KOS, 27US11, 27m152, 27gfp, and 27gfpR was digested with the indicated restriction enzymes and probed for the junction of HSV UL26–UL27. The predicted band sizes are indicated in the tabular inset.

We generated rHSVs expressing HCMV US11 (27US11), MCMV m152 (27m152), or a mock recombinant expressing only the *gfp/gpt* selection cassette (27gfp) as described in Materials and Methods and shown schematically in Figure 1A. Proper homologous recombination was confirmed by Southern blots for *gpt,* US11, m152, or the UL26–UL27 junction region. Insertion of the *gfp* selection cassette resulted in a 2.4-kB band shift compared to the parental KOS strain, while selection cassettes containing US11 or m152 gave band shifts of 3.8 kB or 4.2 kB, respectively (Figure 1B). These corresponded with the predicted band sizes indicating correct targeting (Figure 1B). The revertant virus generated from 27gfp (27gfpR) appeared identical to KOS. All viral genomic DNA hybridized with the HSV 26–27 probe as shown. Conversely, only 27gfp, 27US11, and 27m152 hybridized with a *gpt* probe, and only 27m152 and 27US11 hybridized with an m152 and US11 probe, respectively (data not shown). Thus all recombinant viruses contain the appropriate genes inserted into the UL26–UL27 junction region.

### 27US11 and 27m152 Specifically Inhibit Murine MHC Class I

**Figure 2:**
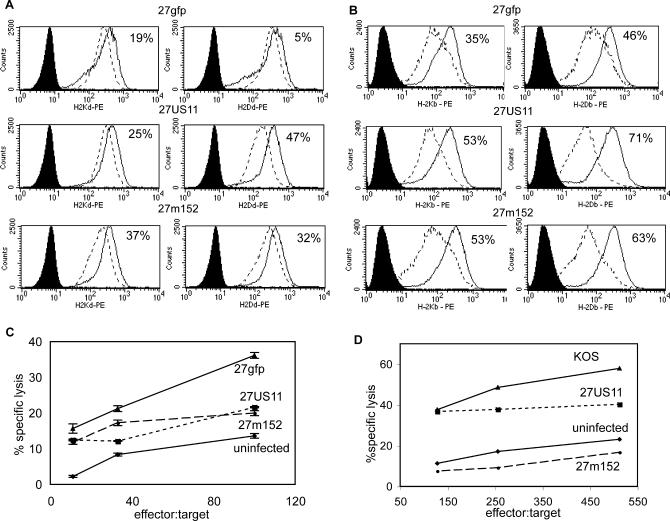
27US11 and 27m152 Inhibit Murine MHC Class I Preventing Lysis by HSV-Specific CTL (A) K-BALB (H-2^d^) and (B) MC57G (H-2^b^) fibroblast cell lines were uninfected (solid line) or infected (dashed line) at an MOI of 5:1 with 27gfp, 27US11, or 27m152 for 18 h and analyzed for surface MHC class I expression. Filled histograms are isotype controls. The percentage reduction in mean fluorescent intensity from uninfected to infected cells is indicated. (C) and (D) Cells were infected as for (A) and (B) and co-incubated with CTL isolated from HSV-infected (C) BALB/c or (D) BALB.B mice at the indicated effector-to-target ratio.

To determine whether 27m152 and 27US11 inhibit surface expression of murine MHC class I more efficiently than the control 27gfp, we analyzed expression on infected murine fibroblasts by flow cytometry. The control 27gfp demonstrated a modest reduction in surface expression of MHC class I 18 h after infection (Figure 2A and 2B), which was similar to the parental KOS strain (data not shown) and consistent with nonspecific vhs-mediated inhibition. Both 27US11 and 27m152 inhibited MHC class I surface expression to a greater extent than the 27gfp control. While 27m152 inhibited all tested murine MHC class I alleles, 27US11 inhibited D^d^, K^b^, and D^b^ but not K^d^, which is consistent with results reported by others [24].

Owing to the slow turnover of MHC class I on the cell surface, total MHC class I expression will underestimate the impact of US11 and m152 on presentation of viral peptides on newly synthesized MHC class I to CD8 T cells. To test directly the effects of rHSVs on recognition of infected targets, we examined the lysis of infected fibroblasts by CTL in vitro. HSV-specific CTL, which efficiently lysed 27gfp-infected fibroblasts, lysed fibroblasts infected with 27US11 or 27m152 less effectively (Figure 2C and 2D). Although, as determined by flow cytometry, overall inhibition of cell-surface MHC class I was greater in the H-2^b^ (Figure 2B) than in the H-2^d^ cells (Figure 2A), the decrease in lysis efficiency of cells infected with 27US11 and 27m152 compared to 27gfp (and KOS, data not shown) was observed both with infected H-2^d^ (Figure 2C) and H-2^b^ (Figure 2D) targets. These data demonstrate that 27US11 and 27m152 have a gain-of-function resulting in increased inhibition of MHC class I antigen presentation and inhibition of CTL-mediated lysis of infected murine cells.

### 27m152, but Not 27US11, Inhibits NKG2D Ligands, Preventing NK-Cell-Mediated Lysis

**Figure 3:**
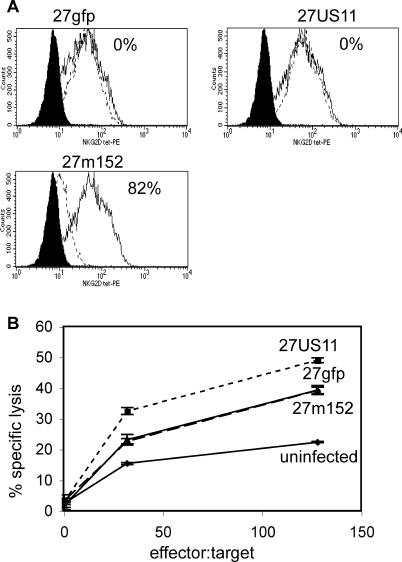
27m152, but Not 27US11, Evades NK Recognition by Inhibiting NKG2D Ligands (A) BALB/c fibroblasts were uninfected (solid line) or infected (dashed line) for 18 h at an MOI of 5:1 with 27gfp, 27US11, or 27m152 (dashed line) and stained with NKG2D tetramer. Solid histogram is uninfected cells stained with an irrelevant tetramer. The percentage reduction in mean fluorescent intensity from uninfected to infected cells is indicated. (B) Cells were prepared as in (A) and co-incubated with splenocytes from naïve RAG1^−/−^ BALB/c mice treated with polyI:C 24 h earlier at the indicated effector-to-target ratio.

Because MHC class I molecules are ligands for inhibitory receptors on NK cells, inhibition of MHC class I surface expression would be predicted to render cells infected with 27US11 and 27m152 more vulnerable to NK-cell-mediated clearance [26]. However, MCMV m152 also inhibits expression of the Rae-1 family of ligands for the NKG2D-activating receptor on NK cells; thus 27m152 should also antagonize NK cell recognition [12,13]. By contrast, HCMV US11 is not known to inhibit the expression of NKG2D ligands, and therefore cells infected with 27US11 should be more vulnerable to lysis by NK cells. Consistent with these predictions, 27m152, but not 27US11, inhibited the expression of NKG2D ligands on infected fibroblasts (Figure 3A), and 27US11-infected fibroblasts were more readily lysed by NK cells than 27gfp-infected fibroblasts. Conversely, lysis of 27m152-infected cells was similar to lysis of cells infected with the control 27gfp virus (Figure 3B). Since the only reported function of HSV ICP47 is to block MHC class I antigen presentation, 27US11 appears to more closely parallel in mice the immune-evasion profile of HSV in humans.

### Recombinant Viruses Grow as Well as Wild-Type in Vitro

**Figure 4:**
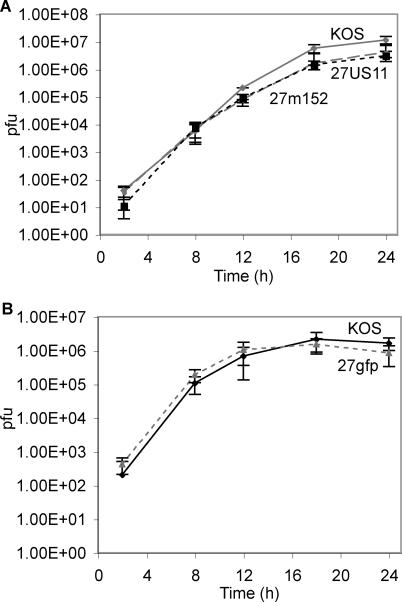
Single-Step Growth Kinetics of rHSVs Are Similar to KOS Vero cells were infected at an MOI of 5:1 with KOS plus (A) 27m152 or 27US11, or (B) 27gfp. Cells and supernatants were harvested at indicated times and viral titers were determined on vero cells.

All three gene products flanking the insertion area (UL26, UL26.5, and UL27) are required for in vitro growth [27]. To confirm that recombination did not alter neighboring gene products, we analyzed the single-step growth kinetics of each virus. Growth curves over a 24-h period revealed that each recombinant virus grew at the same rate as the parent virus (Figure 4A and 4B). Thus, growth of recombinant viruses is not impaired, demonstrating that no genes essential to in vitro growth, including UL26, UL26.5, and UL27, were altered. This finding was confirmed by quantitative RT-PCR for UL26, UL26.5, and UL27 mRNA. Expression for each gene was similar for all three recombinant viruses and for KOS virus (data not shown); expression of GFP and GPT was also similar for all three recombinant viruses.

### The *gfp*-Containing Selection Cassette Attenuates Recombinant Viruses in Vivo

**Figure 5:**
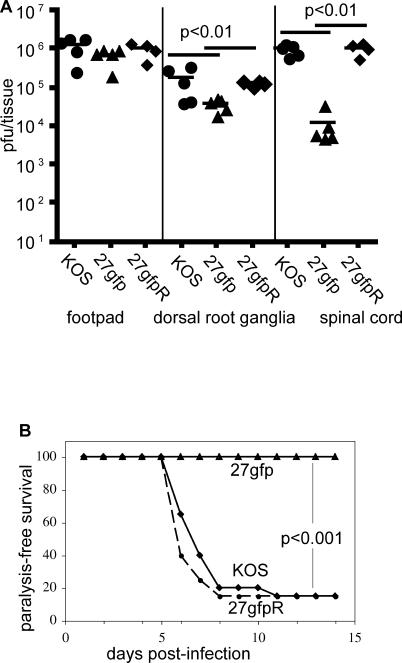
The Selection Cassette Attenuates Neuroinvasiveness and Neurovirulence BALB/c mice were infected in the hind footpads with 2.5 × 10^5^ pfu of KOS, 27gfp, or 27gfpR. (A) The indicated tissues were isolated on day 6, homogenized, and viral titers were determined on vero cells. (B) Ten (27gfp) or 20 (KOS and 27gfpR) mice per virus were monitored for paralysis induction for 14 d. Mice displaying ataxia or paralysis were euthanized.

Recently, Halford et al. reported a KOS-strain rHSV expressing *gfp* driven by the HCMV immediate early promoter inserted between UL26 and UL27 [28]. This virus demonstrated a 50% increase in time-to-death after ocular infection of *scid* mice of C57Bl/6 or BALB/c background. To determine whether the *gfp/gpt* selection cassette used to generate 27gfp, 27US11, and 27m152 resulted in a similar attenuation in vivo, we compared the neuroinvasiveness and neurovirulence of 27gfp to the parental KOS strain and the revertant 27gfpR. Six days post-infection, viral titers from the footpad, dorsal root ganglia, and spinal cord were similar between BALB/c mice infected with KOS or 27gfpR. Although the viral burden in the footpads of 27gfp-infected mice was similar to KOS- or 27gfpR-infected mice, there was a ~10-fold reduction of virus in the dorsal root ganglia and ~100-fold reduction of virus in the spinal cord (Figure 5A). Thus, insertion of the selection cassette reduced neuroinvasiveness, while removal of the cassette restored it. This decrease in neuroinvasiveness correlated with decreased neurovirulence, as 80% of mice infected with KOS or 27gfpR succumbed to paralysis by day 10, while all mice infected with 27gfp retained full mobility (Figure 5B). As the revertant is identical to the parental virus in both neuroinvasiveness and neurovirulence, and 27US11 and 27m152 contain this selection cassette, we used 27gfp as a control to determine the effects of MHC class I inhibition by 27US11 or 27m152 in vivo.

### Inhibition of MHC Class I Is a Virulence Factor

**Figure 6:**
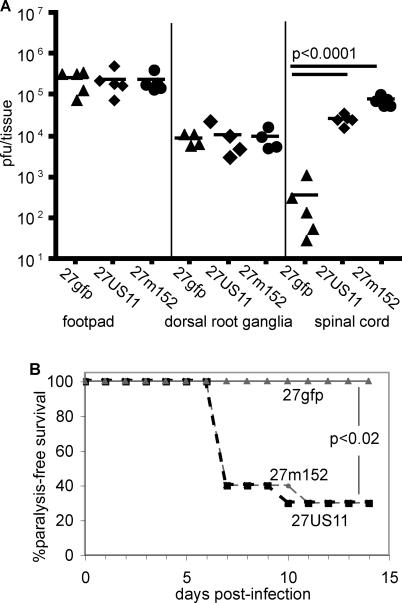
Inhibition of MHC Class I Increases Neuroinvasiveness and Neurovirulence BALB/c were infected in the hind footpads with 2.5 × 10^5^ pfu of 27gfp, 27US11, or 27m152. (A) The indicated tissues were isolated on day 6, homogenized, and viral titers were determined on vero cells. (B) Ten mice per virus were monitored for paralysis induction for 14 d. Mice displaying ataxia or paralysis were euthanized.

There was no difference in the viral burden in the dorsal root ganglia or hind footpad of BALB/c mice inoculated with 27gfp, 27US11, or 27m152. However ~100-fold more virus was recovered from the spinal cord of mice infected with 27US11 or 27m152 compared to 27gfp (Figure 6A). Consistent with this increase in neuroinvasiveness, 27US11 and 27m152 induced paralysis in 70% of mice, while 27gfp did not induce paralysis with this inoculum (Figure 6B). Similar results were obtained in BALB.B mice (data not shown).

### Increased Neurovirulence Is Due to Inhibition of MHC Class I Antigen Presentation to CD8 T Cells

**Figure 7:**
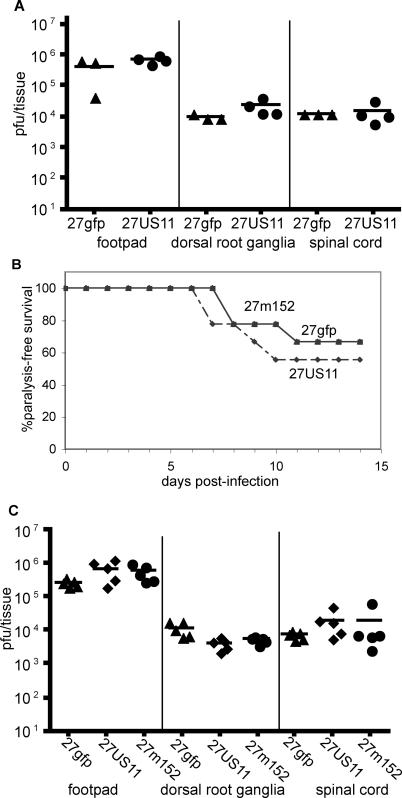
Differences in Neuroinvasiveness and Neurovirulence Are Dependent on MHC Class I and CD8 T Cells β_2_m^−/−^ BALB/c mice were infected in the hind footpads with 3.0 × 10^4^ pfu of 27gfp, 27US11, or 27m152. Note that a lower inoculum was used in these experiments with β_2_m^−/−^ mice than in wild-type BALB/c mice shown in other figures. (A) The indicated tissues were isolated on day 6, homogenized, and viral titers were determined on vero cells. (B) Nine mice per virus were monitored for paralysis induction for 14 d. Mice displaying ataxia or paralysis were euthanized. (C) BALB/c mice were depleted of CD8 cells and infected with 2.5 × 10^5^ pfu of the indicated virus in the hind footpads. Viral titers were determined on day 6.

If these differences in neuroinvasiveness and neurovirulence are solely due to altered antigen presentation to CD8 T cells by MHC class I on infected cells, then 27gfp should be as neurovirulent as 27m152 or 27US11 in mice lacking MHC class I expression and in mice depleted of CD8 T cells. Consistent with this prediction and in sharp contrast to findings in wild-type mice (Figure 6), titers of 27gfp in the footpad, dorsal root ganglia, and spinal cord of β_2_m^−/−^ mice at day 6 were equivalent to those for 27US11 (Figure 7A), and the frequency of paralysis in MHC class I-deficient β_2_m^−/−^ mice infected with 27gfp, 27US11, or 27m152 was similar (Figure 7B). Moreover, the titers of 27gfp in the footpad, dorsal root ganglia, and spinal cord of wild-type mice depleted of CD8 T cells were equivalent to those for 27US11 and 27m152 on day 6 (Figure 7C). Similar findings were obtained at earlier time points—viral burdens in the spinal cord on day 4 were already significantly higher in mice infected with 27US11 compared to mice infected with 27gfp, and this difference was also abolished by CD8 depletion (data not shown). Conversely, depletion of CD4 T cells did not abolish the increase in viral load in the spinal cord of 27US11- or 27m152-infected BALB/c, as compared to 27gfp-infected mice (Figure S1). These findings indicate that HSV-specific CD8 T cells are controlling 27gfp, but not 27US11 or 27m152, and are consistent with the notion that the increased neurovirulence and neuroinvasiveness of 27US11 and 27m152 rHSV are attributable to MHC class I inhibition and evasion of CD8 T cells.

### MHC Class I Inhibition Does Not Affect the Numbers of HSV-Specific CD8 T Cells

The increased neurovirulence of 27US11 and 27m152 could result from impaired generation of HSV-specific CD8 T cells, impaired recognition of infected cells in neural tissues, or both. Recent work suggests that the generation of HSV-specific CD8 T cells in mice infected with wild-type HSV relies on cross-presentation of viral antigens by dendritic cells [29]. If this is the case, increased MHC class I inhibition by 27US11 and 27m152 should not affect the induction of HSV-specific CD8 T cells.

**Figure 8:**
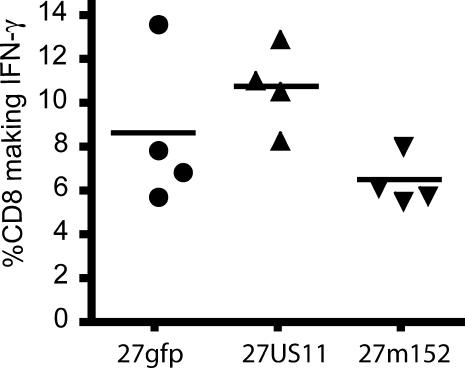
The Size of the CD8 T-Cell Response to rHSVs Is Not Altered by MHC Class I Inhibition Lymphocytes from the draining popliteal lymph nodes were isolated from BALB.B mice on day 6 of infection with 27gfp, 27US11, or 27m152. Lymphocytes were stimulated with HSV gB_498–505_ for 5 h then stained for CD8 and intracellular IFN-γ. Of the unstimulated CD8^+^ cells, <0.1% were IFN-γ^+^.

To address this, we infected BALB.B mice, which are congenic with BALB/c mice, and similar in their susceptibility to HSV, but of the H-2^b^ haplotype. Since ~90% of HSV-specific CD8 T cells in H-2^b^ mice recognize the peptide gB_498–505_ presented by H-2K^b^, BALB.B mice allow accurate enumeration of antigen-specific CD8 T cells [30]. At the peak of infection, 27US11, 27m152, and 27gfp induced similar numbers of gB-specific IFN-γ-producing CD8 T cells (Figure 8). Thus, the difference in neuroinvasiveness and neurovirulence between these viruses was not due to altered generation of gB-specific CD8 T cells.

## Discussion

In this report, we show that two different rHSVs, 27US11 and 27m152, efficiently inhibited antigen presentation by MHC class I molecules on murine cells, as does wild-type HSV on human cells. BALB/c and BALB.B mice infected with either of these two rHSVs showed an increased incidence of paralysis induction in vivo compared to mice infected with the control 27gfp virus. Paralysis induction correlated with higher viral burden in the CNS, but not in the footpad or the peripheral nervous system. This increase in neurovirulence occurred despite the presence of a strong antigen-specific CD8 T-cell response, the size of which was not diminished in mice infected with 27US11 or 27m152 compared to 27gfp. By contrast, increased neurovirulence of 27US11 and 27m152 did correlate with the reduced sensitivity of target cells infected with these viruses to CD8 CTL lysis in vitro*.* The greater neurovirulence of 27US11 and 27m152 was not observed in MHC class I-deficient mice or in wild-type mice depleted of CD8 T cells. Taken together, these data indicate that inhibition of MHC class I antigen presentation by HSV is a neurovirulence factor, and that the primary mechanism for this increased virulence was the inhibition of target-cell recognition by antigen-specific CD8 T cells.

A previous study addressed the importance of ICP47-mediated inhibition of MHC class I antigen presentation by HSV in mice [23]. When HSV-susceptible A/J or BALB/c mice were challenged by ocular inoculation with a mutant HSV lacking ICP47, this strain induced a lower incidence of neurologic symptoms and death than the parental strain F. This difference was ablated when mice were depleted of CD8 cells and in mice that lacked CD8 T cells. This study suggested that although ICP47 has a much reduced impact on TAP-dependent antigenic peptide transport in murine cells versus human cells, this may be sufficient for functional inhibition in mice in vivo. However, the relatively weak effect of ICP47 in murine cells may underestimate its importance in viral pathogenesis in humans [21,22]. Using rHSVs that more closely approximate in mice the magnitude of MHC class I inhibition by wild-type HSV in humans, the impact of MHC class I inhibition on neurological outcome was clear—the titers of 27US11 and 27m152 were more than 100-fold higher, and the frequency of paralysis was significantly greater than 27gfp.

In the previous study, no attempt was made to determine whether the difference in neurological outcome was associated with differences in viral titers in peripheral tissues or the CNS or with differences in the generation of antigen-specific CD8 T cells [23]. We found that, despite robust inhibition of antigen presentation by murine MHC class I, the only significant difference in viral titers was found in the CNS, suggesting a focused role for this evasion strategy. This focused immune evasion may result from the greater impact of inhibition of MHC class I antigen presentation on cells that express low levels of MHC class I, such as neurons during acute infection, making the infected CNS more susceptible to this immune-evasion strategy [31].

Inhibition of antigen presentation on MHC class I may impact CD8 T-cell response to HSV infection at either or both of two distinct phases. First, inhibition could limit the size of the antigen-specific CD8 T-cell response. However, we found that the frequency of HSV-specific CD8 T cells was not diminished in response to infection with rHSVs that effectively inhibited MHC class I antigen presentation. Our findings provide strong support for the notion that CD8 T-cell priming in mice infected with HSV is carried out by cross-priming, as proposed by others from studies with wild-type HSV [29,32,33]. The second phase at which inhibition may impact outcome is at the site of productive infection, in the peripheral tissues, peripheral nervous system, or CNS. The selective increase in viral titers in the CNS in mice infected with 27US11 and 27m152 in the absence of differences in the magnitude of the CD8 T-cell response is compatible with the notion that the primary effect of MHC class I inhibition by HSV is to prevent recognition of infected cells by virus-specific CD8 T cells in the CNS. Thus, generation of an immune response does not always predict the functional relevance of that response. This distinction is important to the evaluation of vaccines targeted at pathogens that specialize in immune evasion such as herpesviruses and poxviruses.

Although depletion of CD8 T cells had a profound impact on the amount of 27gfp in the spinal cord, there was little difference in titers of any of the rHSVs in the footpad and dorsal root ganglia between intact and depleted mice (see Figures 6A and 7C). This result suggests that the primary impact of CD8 T-cell immunity to HSV is in the CNS. That depletion of CD8 T cells in mice infected with 27US11 or 27m152 did not affect CNS viral titers indicates that the effect of MHC class I inhibition is also manifest primarily at this site.

The effect of m152 expression in HSV on viral titer is different from the effect of m152 in MCMV. As reported by others, deletion of m152, together with the other MHC class I inhibitors expressed by MCMV, m04 and m06, has no effect on viral titers in the lung [34]. This difference may be due to the different tropisms of HSV and MCMV. Whereas MCMV infects cells with moderate-to-high expression of MHC class I, HSV targets neuronal cells which express very little MHC class I [31], and the impact of MHC class I inhibition would be more apparent in cells that normally express low levels of MHC class I. This difference may explain the significant increase in viral titer in the CNS of 27m152- and 27US11-infected mice, while lung titers of wild-type and Δ04+Δ06+Δ152 MCMV are similar.

While the outcome of infection with 27US11 and 27m152 viruses in vivo was similar, and these viruses inhibited CD8 T-cell-mediated killing of infected cells in vitro to a similar degree, cells infected with 27US11 but not 27m152 were more susceptible to killing by NK cells in vitro. Together, these findings suggest that evasion of NK cells does not substantially impact the outcome of acute HSV infection when CD8 T-cell recognition is impaired in mice of the susceptible BALB background. Furthermore, depletion of NK cells with anti-asialoGM1 antiserum did not abrogate the difference in viral titers in the CNS in mice infected with 27US11 and 27m152 compared to 27gfp—on day 3, titers of 27US11 and 27m152 were similar, and both were significantly greater than the titer of 27gfp (data not shown). It is possible that this finding may be mouse-strain-specific, as is the case for m152-mediated NK cell evasion for MCMV [13].

Another limitation of traditional murine models of HSV infection is the lack of spontaneous reactivation in vivo*,* which is a hallmark of human infection. This may reflect the fact that latently infected mice maintain HSV-specific CD8 T cells in the infected ganglia, which can prevent reactivation of wild-type HSV in vitro [35]. Given the greater efficiency with which they evade CD8 recognition, it is possible that 27US11 and 27m152 may display altered latency features in mice compared to wild-type HSV.

## Materials and Methods

### Cell lines and mice.

Vero cells were used for isolation of recombinant viruses. Viral stocks were prepared from infected vero cells at 90%–100% cytopathic effect. Stocks were sonicated, stored at −80 °C, and titered on vero cells. Plaque formation was visualized with crystal violet stain in 10% formaldehyde. Revertant virus was isolated from STO cells (ATCC, Manassas, Virginia, United States) that lack HPRT making them resistant to 6-thioguanine toxicity. Murine fibroblast cell lines (ATCC) MC57G from C57Bl/6 and K-BALB from BALB/c mice were used to assess MHC class I expression and CD8 T-cell- and NK-cell-mediated lysis.

Female BALB/c and Rag1^−/−^ (BALB/c background) were purchased from Jackson Laboratory (Bar Harbor, Maine, United States) and used at 6–8 wk of age. BALB.B and β_2_m^−/−^ (BALB/c background) breeder pairs were purchased from Jackson Laboratory and bred in-house. All mice were maintained in the University of Washington SPF facility. All studies were approved by the University of Washington Animal Care and Use Committee.

### Generation of rHSV*.*

To determine the effect of MHC class I inhibition on HSV infection in mice, we generated rHSVs that express MCMV m152 or HCMV US11, denoted 27m152 and 27US11, respectively (see Figure 1). Additionally, a control virus termed 27gfp, which expresses the selection cassette used to isolate 27m152 and 27US11, was generated. A 2.5-kB EcoR I–Hind III fragment in the UL26–UL27 region of the HSV genome was isolated and cloned into pUC19. A unique Not I site in the non-coding region that separates the UL26 and UL27 open reading frames was mutated to a Spe I site and used for generation of the targeting vector. A selection cassette containing *eGfp* driven by the EF-1 promoter and *E. coli* guanosylphosoribosyltransferase *(gpt)* driven by the PGK-1 promoter was cloned into the Spe I site. The original Not I site is located in the polyA signal of UL26. To correct for this, the bi-directional SV40 polyA was also included. This targeting vector was used to generate 27gfp via homologous recombination.

The targeting vector was digested with EcoR I and Hind III, and 10 μg of DNA was electroporated into vero cells using a Bio-Rad (Hercules, California, United States) GenePulser Xcell (273 V, 1,100 μF, 186 Ω, and 0.4-mm cuvette). Electroporated cells were plated in six well plates and infected with 2.5 × 10^5^ pfu KOS strain HSV-1 24 h after electroporation.

Similarly, 27US11 and 27m152 were generated by inserting HCMV US11 or MCMV m152 into the *gfp/gpt*-targeting vector under the control of the HCMV IE promoter. MCMV m152 was PCR-amplified from viral genomic DNA, while HCMV US11 was isolated from an expression vector (provided by Stan Riddell, Fred Hutchinson Cancer Research Center). Recombinant viruses were plaque-purified based on gfp expression, passed once through BALB/c mice, and re-isolated from the spinal cord to ensure neuroinvasiveness. A revertant virus for 27gfp, termed 27gfpR, was generated via homologous recombinant in STO cells and isolated by loss of gfp expression. Isolation of the revertant virus was supported by negative selection in the presence of 40 μg/ml 6-thioguanine, which is converted to the toxic 6-thioxanthine by GPT [36].

### MHC class I and NKG2D surface expression.

MC57G (H-2^b^) or K-BALB (H-2^d^) fibroblast cell lines were infected at a multiplicity of infection (MOI) of 5:1 for 18 h in the presence of 200 μM gancyclovir. Cells were resuspended in 100 μl of phosphate-buffered saline + 1% bovine albumin and 0.09% sodium azide with α-CD16/32 (1:100) as an Fc-blocking reagent and phycoerythrin-conjugated α-H-2K^b^ (1:100), α-H-2D^b^ (1:25), α-H-2K^d^ (1:100), α-H-2D^d^ (1:100), muIgG_2a_κ and muIgG_2b_κ isotype controls, NKG2D tetramer (1:1000), or irrelevant tetramer (1:1,000) for 30 min on ice. Phycoerythrin-conjugated tetramers were produced as previously described [37]. Analysis was performed with a Becton Dickenson (Palo Alto, California, United States) FACSscan. Antibodies were purchased from BD Biosciences Pharmingen (San Diego, California, United States).

### CTL and NK lysis*.*

MC57G or K-BALB cells were infected at an MOI of 5:1 for 11 h (for CTL assays) or for 8 h (for NK cell assays) in the presence of 200 μM gancyclovir. Cells were resuspended in 300 μl of warm media with 30 μl of fresh ^51^Cr (PerkinElmer, las.perkinelmer.com). Cells were incubated at 37 °C for two 1-h periods and washed twice with warm media. Co-incubation of 10^5^ cells/well took place for 5 h (CTL) or 8 h (NK) with effector cells. Eighteen hours after infection, 100 μl of media was collected and analyzed on a Wallac 1470 Wizard gamma counter (PerkinElmer). CD8 CTLs were derived from the draining lymph node of day-6 HSV-infected BALB.B or BALB/c mice. For 6 d, 10^6^ lymphocytes were cultured in DMEM (Gibco, San Diego, California, United States) plus 10% FBS with 1:100 anti-CD3 and 25 μg/ml recombinant huIL-2. Media was changed on days 3 and 5 with fresh huIL-2. These CTL were added to target cells in graded numbers. Activated NK cells were derived from splenocytes from Rag1^−/−^ BALB/c mice injected i.p. with 200 μg polyI:C 24 h prior to sacrifice. Total splenocytes were added to target cells in graded numbers. Specific lysis was determined: percentage specific lysis = (count − minimum)/(total lysis − minimum lysis) × 100.

### Single-step growth in vitro.

Vero cells were infected at an MOI of 5:1 for 1 h at 4 °C to allow viral attachment. Cells were then washed thrice with cold PBS and warm RPMI media with 10% FBS added (*t* = 0). Cells were incubated at 37 °C for 1 h. Media was then removed and cells were quickly washed twice with sodium citrate buffer (pH 3.0) and rinsed thrice with warm PBS. Warm media was then replaced and cells were returned to 37 °C. At indicated time points, media from three wells per virus was collected; cells were then trypsinized and mixed with the corresponding media. Samples were stored at −80 °C and titered on vero cells.

### Infection of mice.

Mice were infected in the hind footpads with the indicated inocula following dermal abrasion, as described previously [38]. In this model, virus travels anterograde up the enervating sciatic nerve to the dorsal root ganglia, replicates in the ganglion, and can then return to the site of infection via retrograde axonal transport resulting in a primary lesion of the footpad. Virus in the dorsal root ganglia can also bridge the synapse and enter the CNS at the spinal cord, from which it may ascend towards the brain. Infected mice were monitored twice daily for 14 d for ataxia and hind-limb paralysis. Previous findings indicated that more than 80% of mice displaying paralysis succumb to infection; thus paralyzed mice were euthanized in accordance with our IACUC protocol.

### In vivo viral titers.

Infected mice were euthanized on day 6 of infection. Hind footpads, dorsal root ganglia with proximal sciatic nerve, and spinal cord were isolated and snap frozen on dry ice. Samples were stored at −80 °C. All samples were homogenized and titered in triplicate on vero cells.

### Antibody depletion.

Mice were depleted of CD4 or CD8 T cells by i.p. injection of 200 μl of 1 mg/ml anti-CD4 (GK1.5) or anti-CD8 (clone 2.43) on two consecutive days, and were infected 2 d later. Mice were depleted of NK cells by i.p. injection of 100 μl of α-asialo-GM1 (Wako Biochemical, http://www.wako-chemicals.de) 1 d before infection.

### Quantification of CD8 T-cell response.

BALB.B mice were infected with 2.5 × 10^5^ pfu of HSV and sacrificed on day 6. Single-cell suspensions were prepared from their draining popliteal lymph nodes. For IFN-γ production, cells were stimulated with 5 μM of the immunodominant HSV peptide glycoprotein B_498–505_ (gB_498–505_) (United Biochemical Research, Seattle, Washington, United States) for 5 h in the presence of BD GolgiStop, followed by surface staining with anti-CD8-FITC (1:100), permeabilized as above and stained for intracellular IFN-γ with anti-IFN-γ-PE (1:200). Unstimulated lymphocytes were used as a negative control. Specific IFN-γ production = percentage CD8^+^ IFN-γ^+^ stimulated − unstimulated. All antibodies were purchased from BD Biosciences Pharmingen.

## Supporting Information

Figure S1 Depletion of CD4 T Cells Does Not Equalize Spinal Cord Titers of 27 gfp Compared to 27US11 or 27m152, Whereas Depletion of CD8 T Cells Does BALB/c mice were either (A) untreated; or (B) and (C) treated 3 and 2 days before the time of infection with 200 μg α-CD4 MAb (GK1.5) or α-CD8 MAb (53–6.7), respectively. Mice were infected with 2.5 × 10^5^ pfu of 27 gfp, 27US11, or 27m152 on day 0, and footpads and spinal cord were harvested on day 6. This experiment was distinct from the experiments shown in Figures 6 and 7, but the findings for A and C are similar to those shown in Figures 6A and 7C, respectively.
(357 KB EPS)

### Accession Numbers

The Swiss-Prot (http://www.ebi.ac.uk/swissprot) accession number for the MCMV m152 gene product is Q69G18, and for the HCMV US11 gene product is P09727. The Swiss-Prot accession number for vhs is Q69G18, and for the US12 gene product ICP47 is P03170. Swiss-Prot accession numbers for UL26, UL26.5, and UL27 are P10210 and P10211.

## Abbreviations

CNS: central nervous system
CTL: cytotoxic T lymphocyte
HCMV: human cytomegalovirus
HSV: herpes simplex virus
MCMV: murine cytomegalovirus
MHC: major histocompatibility complex
MOI: multiplicity of infection
NK: natural killer
rHSV: recombinant herpes simplex virus
TAP: transporter associated with antigen presentation
